# A Multilevel Analysis of Determinants of Dying in Place among Assisted Living Residents

**DOI:** 10.1093/geroni/igaf122.702

**Published:** 2025-12-31

**Authors:** Emmanuelle Belanger, Xiao (Joyce) Wang, Melissa Clark, Joan Teno, Kali Thomas, Pedro Gozalo

**Affiliations:** Brown University, Providence, Rhode Island, United States; Brown University, Providence, Rhode Island, United States; Brown University, Providence, Rhode Island, United States; Brown University, Providence, Rhode Island, United States; Johns Hopkins University, Baltimore, Maryland, United States; Brown University, Providence, Rhode Island, United States

## Abstract

Many older adults residing in assisted living (AL) consider this setting their home; yet, there is limited evidence about determinants of their ability to die in place, with state regulations explaining only some variation in this outcome. We examined the likelihood of residents dying in place as a function of state regulations and AL-community factors. To do this, we combined an inventory of state regulations with a nationally-representative survey of over 2,000 AL administrators from 48 states about care processes, and claims data for 9,803 deceased fee-for-service Medicare beneficiaries in 2021-2022. Variables included state regulations related to third-party services (e.g. license explicitly allows hospice), survey variables about organizational characteristics (size, neighboring facilities, for-profit status, accepting Medicaid), care processes (staffing, quality of collaboration with hospice), resident characteristics (demographics, chronic conditions) and a geographic (HSA) indicator of intensity of end-of-life care. Multilevel models were estimated with sampling weights to obtain nationally-representative estimates. State regulations were not significantly associated with dying in place in fully adjusted models. Residents were more likely to die in place when residing in AL communities with for-profit status and those that did not accept Medicaid. Residents at communities near a nursing home were significantly less likely to die in place. In terms of care processes, dying in place was associated with administrators arranging for hospice care (rather than referring out), and reporting higher quality of collaboration with hospice providers. AL characteristics and care processes are more important determinants of dying in place than state regulations.

